# Effects of a TREM-like transcript-1 derived peptide during septic shock in pigs

**DOI:** 10.1186/cc11735

**Published:** 2012-11-14

**Authors:** S Gibot, A Boufenzer, Y Bouazza, F Groubatch, C Alauzet, D Barraud, PE Bollaert, P Leroy, N Tran, M Derive

**Affiliations:** 1 CHU Nancy, Nancy, France; 2 Nancy University, Groupe CHOC - Inserm U961, Nancy, France; 3 Nancy University, School of Surgery - U961, Nancy, France; 4 Nancy University, EA4369, Nancy, France; 5 Nancy University, EA3452 CITHEFOR, Nancy, France

## Background

Triggering receptor expressed on myeloid cells-1 (TREM-1) is expressed on innate immune cells and plays a crucial role during the onset of sepsis by amplifying the host immune response. TREM-like transcript-1 (TLT-1) belongs to the TREM family and is selectively expressed on activated platelets. We recently showed that TLT-1 and a TLT-1-derived peptide (LR12) exhibit anti-inflammatory properties by dampening TREM-1 signalling and thus behave as naturally occurring TREM-1 inhibitors [1]. We also, however, demonstrated that the same peptide modulates *in vivo *the inflammatory cascade triggered by infection, thus inhibiting hyper-responsiveness, organ damage and death during sepsis in mice. As mouse models of septic shock are far from recapitulating the human physiology, we investigated the effects of LR12 during peritonitis in adult mini-pigs. Here we show that sepsis-induced cardiovascular dysfunction and organ failure was prevented by LR12 administration. The objective was to determine the effects of a TLT-1 derived peptide (LR12) administration during septic shock in pigs (13 adult male mini-pigs).

## Methods

Two hours after induction of a fecal peritonitis, anesthetized and mechanically ventilated mini-pigs were randomized to receive LR12 (*n *= 6) or its vehicle alone (normal saline, *n *= 5). Two animals were operated and instrumented without the induction of peritonitis and served as controls (sham). Resuscitation was achieved using hydroxyethyl starch (up to 20 ml/kg) and norepinephrine infusion (up to 10 μg/kg/minute).

## Results

**Figure 1:**
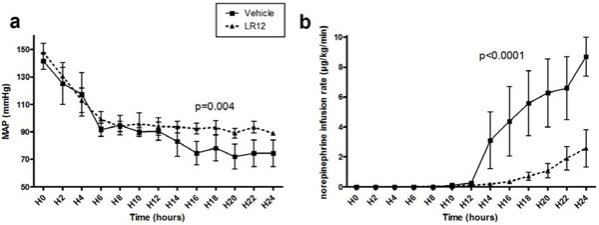
**LR12 protects from sepsis-induced hypotension**. Evolution of mean arterial pressure **(a) **and norepinephrine requirements **(b) **during the 24-hour study period. MAP was constantly higher and norepinephrine dose lower in LR12-treated animals than controls.

Hemodynamic parameters were continuously recorded. Gas exchange, acid-base status, organ function, and cytokines were measured at regular intervals until 24 hours after the onset of peritonitis when animals were sacrificed under anesthesia. Peritonitis induced profound hypotension, myocardial dysfunction, lactic acidosis, coagulation abnormalities, and multiple organ failure. These disorders were largely attenuated by LR12. In particular, cardiovascular failure was prevented as attested by better mean arterial pressure, cardiac index, cardiac power index, and SvO_2_, despite lower norepinephrine requirements (Figure 1). Finally, 24-hour mortality rates were respectively 60% and 0% for control and LR12 groups.

## Conclusion

LR12, a TLT-1 derived peptide, exhibits salutary properties during septic shock in adult mini-pigs.

## References

[B1] Derive M Bouazza Y Sennoun N Marchionni S Quigley L Washington V Massin F Max JP Ford J Alauzet C Levy B McVicar DW Gibot S Soluble TREM-like transcript-1 regulates leukocyte activation and controls microbial sepsis J Immunol 2012 188 5585 5592 10.4049/jimmunol.1102674 22551551 PMC6382278

